# SEVENTH ANNUAL MEDICLINIC MIDDLE EAST RESEARCH CONFERENCE 23 SEPTEMBER 2024

**DOI:** 10.1097/MD.0000000000041417

**Published:** 2025-03-07

## MEDICLINIC MIDDLE EAST ANNUAL RESEARCH CONFERENCE

It takes a team to make discoveries but Mediclinic Middle East has taken this concept well beyond the traditional partnership between students, scientists and research support staff with its expertise in data research, project management, testing, and clinical trials. Mediclinic Middle East Research office provides the highest international ethical and scientific standards in clinical trial operations. Our patients still need the largest amount of research we can do, so that we can continue to provide them with new interventions. After all, that is why our trial patients and their families know they can count on Mediclinic Middle East. No matter what we face, our team, will do whatever it takes to achieve the extraordinary. We will address obstacles, use them as opportunities to meet the needs of our patients and chart a path forward in unimaginable ways – all in the collective pursuit of our mission to improve the quality of healthcare.

Mediclinic Middle East Research office was established in 2016 under the leadership of Dr Pietie Loubser. Its main focus was supporting clinical research in areas that had, historically, been less well researched than cancer and cardiovascular diseases. Now operational for 8 years, 220 research studies are registered with the MCME Research office. Out of these, 41% are Investigator Initiated research studies, 36% are clinical trials sponsored by various biotech and biopharmaceutical industries, 20% research studies are university collaborations/research studies led by students whereas grants and consortium make up only 3% of the total. There were a total of 91 research publications in high indexed journals from MCME in the year 2023.

Mediclinic Middle East established a local Research Ethics Committee that comprises of multi-disciplinary membership of health care professionals that meet once a month to evaluate risks versus benefits ratio of each research proposal and issue decisions to allow for a formal initiation of the research study. This committee was delighted to be recognized as one of the most active research ethics committees at the Department of Health, Abu Dhabi’s first Annual Research Day which was held in December 2023, and this award is a testament to the Research Ethics Committee’s commitment to the highest standards of research within the company. We have also established a separate group called ‘Mediclinic Middle East Research Advisory Group’ with the aim to promote and provide guidance, and prioritize research on unit level. The members of research advisory group streamline research communication within their clusters and advice regarding available research opportunities. This group also meets once a month to discuss research promotion strategy at MCME.

MCME Research office continues to be a thriving department, collaborating with health care professionals both local, nationally and internationally to deliver high quality research. The team has established strengths in breast cancer research, endocrinology, cardiovascular disease, dermatology and neuroscience. It also has a strong research quality management system monitoring the data quality and data protection principles and ensuring safety of the population being studied.

We proudly host the Annual Mediclinic Middle East Research Conference that was introduced in 2018, then a one day event called the Mediclinic Middle East Annual Research Day. In 2018, the Dr. Edwin Hertzog Award in the name of the founding Chair of Mediclinic International, himself a physician with a keen interest in research, was introduced for the best researcher of the year. The award comprises of a floating trophy, a medal and a cash prize. The event has become an important annual event not only as part of the Mediclinic Middle East events calendar but that of the UAE. In 2019, the meeting was opened to the public to attend, by invitation only, but participation by presentation was not allowed. The presentations were also accepted and published by Wolter’s Kluwer as conference proceedings, and have already been published. The platform was opened to audience external to MCME in the year 2019 and thus the change of name to a ‘conference’. The aim of this conference is to showcase the clinical research undertaken at Mediclinic Middle East, as the company leadership strongly believes in clinical research being the cornerstones of progress and innovation in healthcare, leading to superior quality of care for the community it serves. This years’ conference features podium and poster presentations with an address from the chief guest and key note speakers. We also have an expert panel of speakers both from the UAE and abroad. There are a total of 8 podium presentations, 17 poster presentations and two keynote speech from Dr Mohamed Al Ameri titled “Emirati Genome Project” and Behtash Bahadur titled “Returning Research Results to study participants”

We would like to thank all the healthcare professionals who the MCME Research office have collaborated with over the years, and also to the staff for all their hard work.

## AGENDA

Monday, September 23, 2024 Fairmont Bab Al Bahr Hotel

Abu Dhabi

**Table:** 

**TIME**	**DESCRIPTION**	**SPEAKER**
09:00 – 09:30	Registration Mini Breakfast & Coffee	
09:30 – 09:40	Introduction to Research at MCME	**Dr. Rakshinda Mujeeb** Research Projects Manager Mediclinic Middle East, Dubai **Ms. Zineb Katache** Research Assistant Mediclinic Middle East, Dubai
09:40 – 09:45	Opening Remarks	**Dr. Adrian Stanley** Chief Academic Officer Mediclinic Middle East, Dubai
09:45 – 09:50	Opening Remarks	**Mr. Hein VanEck** Chief Executive Officer Mediclinic Middle East, Dubai
09:50 – 09:55	Introduction Address by Guest of Honor	**H.E Dr Asma Ibrahim Al Mannaei** Executive Director of Research & Innovation Department of Health, Abu Dhabi
09:55 – 10:00	Introduction to the Keynote Speaker	**Dr. Glen Hagemann** Director Precision Medicine and Wellness Mediclinic Middle East, Dubai
10:00 – 10:30	**KEYNOTE SPEAKER:** Emirati Genome project	**Dr. Mohamed Alameri** Section Head of Studies and Special Projects Department of Health, Abu Dhabi
10:30 – 10:40	Q & A	
10:40 – 11:15	Coffee & Networking Break	
**Podium Presentation – 1**^**st**^ **Session**		**Dr. Ramzy Ross** **Director Shared Clinical Services** **Mediclinic Middle East, Dubai**
11:15 – 11:30	Evaluating the potential of Vitamin D and curcumin to alleviate inflammation and mitigate the progression of osteoarthritis through their effects on human chondrocytes: A proof-of-concept investigation.	**Ms. Sharon Nandkishore** 5th year Medical Student Mohammed Bin Rashid University of Medicine and Health Sciences (MBRU), Dubai.
11:30 – 11:45	Inactivation of Staph. Epidermidis by iron chelotor Deferoxamine mesylate (DFO) compared with novel iron chelator Starch conjugated DFO (S-DFO)	**Dr. Ibrahim Mustafa** Assistant Professor Faculty of Health Science, Higher Colleges of Technology, Dubai.
11:45 - 11:55	Q & A	
**Podium Presentation – 2**^**nd**^ **Session**		**Ms. Cora Lessing** **Nursing Director** **Mediclinic Airport Road Hospital, Abu Dhabi**
11:55 – 12:10	Emergency Nursing Didactics – Opinions, Resource, and Skill Exchange (E.N.D.O.R.S.E) – A collaborative academic initiative in the Emergency Department	**Dr. Mohammad Anzal Rehman** Specialist Emergency Physician Mediclinic City Hospital, Dubai.
12:10 – 12:25	In-Patient Diabetes Care Link Nurse Team: The Effect of Health Education on Patient Glycaemic Control during Hospitalization and Post-Discharge	**Ms. Wafa Harbaji** Diabetes Educator Mediclinic Airport Road Hospital, Abu Dhabi.
12:55 – 13:10	Factors Influencing the Activation of Code Blue in Nurses and Ways to Improve Nurses **Satisfaction**	**Ms. Shazia Naz** Unit Manager Mediclinic Al Jowhara Hospital, Al Ain.
13:10 – 13:20	Q & A	
13:20 – 14:40	Lunch & Prayer Break	
**Podium Presentation – 3**^**rd**^ **Session**		**Dr. Maryam Al Saeed** **Consultant Endocrinologist** **Mediclinic Dubai Mall**
14:40 – 14:55	Professionalism-training in undergraduate medical education in a multi-cultural, multi-ethnic setting in the Gulf Region: an exploration of reflective essays	**Dr. Rasha Buhumaid** Vice Dean of Graduate Medical Education Mohammed Bin Rashid University of Medicine and Health Sciences (MBRU), Dubai.
14:55 – 15:10	Retrospective Analysis of Management and Outcome of Preterm Premature Rupture of Membranes (PPROM) in Mediclinic Al Noor Hospital.	**Dr. Hala Abdeldafie** Medical Intern, Mediclinic Al Noor Hospital, Abu Dhabi.
15:10 – 15:25	Characterization of the expression of pain receptors in a preclinical model of statin induced myalgia	**Dr. Asha Caroline Cyril** Post-Doctoral Research Fellow Mohammed Bin Rashid University of Medicine and Health Sciences (MBRU), Dubai.
15:25 – 15:35	Q & A	
15:35 – 15:40	Introduction to the Keynote Speaker	**Dr. Adrian Stanley** Chief Academic Officer Mediclinic Middle East, Dubai
15:40 – 16:10	**KEYNOTE SPEAKER:** Sharing Clinical Research Results for Patients and the Public	**Behtash Bahadur** Associate Director, Health Literacy The Center for Information and Study on Clinical Research Participation, Boston MA
16:10 – 16:20	Q & A	
16:20 – 16:30	Closing Remarks and Winner Announcement	**Dr. Pietie Loubser** Chief Clinical Officer Mediclinic Middle East, Dubai **Dr. Adrian Stanley** Chief Academic Officer Mediclinic Middle East, Dubai

## PODIUM PRESENTATION ABSTRACTS

### Evaluating the potential of Vitamin D and curcumin to alleviate inflammation and mitigate the progression of osteoarthritis through their effects on human chondrocytes: A proof-of-concept investigation.: Presenting Author: Dr. Sharon Nandkishore

5th year Medical Student Mohammed Bin Rashid University of Medicine and Health Sciences (MBRU), Dubai.

Sharon Nandkishore^1*^, Rajashree Patnaik^1^, Sumbal Riaz^2^, Bala Mohan Sivani^2^, Shemima Faisal^2^, Nerissa Naidoo^2^ and Yajnavalka Banerjee^1,4^

^*1*^ *Banerjee Research group, College of Medicine and Health Sciences, Mohammed Bin Rashid University of Medicine and Health Sciences (MBRU), Dubai, United Arab Emirates.*

^*2*^ *College of Medicine and Health Sciences, Mohammed Bin Rashid University of Medicine and Health Sciences (MBRU), Dubai, United Arab Emirates.*

^*4*^ *Centre for Medical Education, University of Dundee, Dundee, United Kingdom*

*Current address: Department of Molecular Biology, Lund University, Lund, Sweden.*

^*^ *Contact details of the Presenting Author: Sharon.Nandkishore@students.mbru.ac.ae TEL.: +971-554548707*

**Abstract**

**Background and Objective:** Osteoarthritis (OA) is a chronic degenerative joint disorder primarily affecting the elderly, characterized by a prominent inflammatory component. The long-term side effects of current therapies necessitate the development of safer and more efficacious alternatives. Nutraceuticals like Vitamin D and curcumin offer potential anti-inflammatory benefits for OA. Variable Vitamin D outcomes and, preliminary curcumin data in OA emphasize the need for further investigation in OA management.

**Methods and Results:** In this study, we utilized a proinflammatory human chondrocyte OA model to assess the anti-inflammatory properties of Vitamin D and curcumin, with a particular focus on the Protease-Activated Receptor-2 (PAR-2) mediated inflammatory pathway. Employing a robust Small Interfering Ribonucleic Acid (siRNA) approach, we modulated PAR-2 expression to understand its role in inflammation.

Results indicate that both Vitamin D and curcumin attenuate PAR-2 expression, leading to a reduction in the downstream proinflammatory cytokines, such as Tumor Necrosis Factor-alpha (TNF-α), Interleukin 6 (IL-6), and Interleukin 8 (IL-8), implicated in the OA. Concurrently, these compounds suppressed the expression of Receptor Activator of Nuclear Factor kappa-Β Ligand (RANKL) and its receptor RANK, which are associated with PAR-2 mediated TNF-α stimulation. Additionally, Vitamin D and curcumin downregulated the expression of Interferon gamma (IFN-γ), underscoring their potential therapeutic implications in OA.

**Conclusions:** This study provides evidence of the mitigating effect of Vitamin D and curcumin on PAR-2 mediated inflammation. Thus, our findings pave the way for future research and the development of novel, safer, and more effective therapeutic strategies for managing OA.

This work is supported by a grant from MBRU given to Drs. Banerjee and Naidoo. Research narrated in this abstract has been published in PLoS One. 2023 Dec 29;18(12):e0290739. doi: 10.1371/journal.pone.0290739.

### Inactivation of Staph. Epidermidis by iron chelotor Deferoxamine mesylate (DFO) compared with novel iron chelator Starch conjugated DFO (S-DFO): Presenting Author: Dr. Ibrahim Mustafa

Assistant Professor, Faculty of Health Science, Higher Colleges of Technology, Dubai.

Ibrahim Mustafa*, Alya Abduljalil Alblooki, Asma Khaled Chehadeh, Fatima Saif Alketbi, Mouza Majed Alsuwaidi, Khadija Rabeea Alnuaimi, Sahar Arafa

*College of Health Science, Higher Colleges of Technology, Sharjah Women’s College, Sharjah, United Arab Emirates*

*Address all correspondence to: imustafa@hct.ac.ae

**Abstract:** Platelet concentrates from apheresis or whole blood obtained have long been contaminated by germs in transfusion medicine. Bacterial contamination of platelets and other blood products causes septic episodes, which have been related to transfusions.

**Objective:** Since platelets are kept at 22 ± 2°C for up to 5 days as opposed to red blood cells (RBC), which are kept at 4°C, there is a possible risk of bacterial contamination. Iron, a crucial element needed for bacterial growth, can also be found in small amounts of RBC that are periodically tainted with platelets. Iron chelators appear to be quite important for reducing bacterial contamination in blood products like platelet bags.

**Methods:** We performed on bacterial growth show that DFO can inhibit *staphylococcus epidermidis* growth, while the addition of 100 μM Fe3+ restores bacterial growth. *S. epidermidis* were exposed for 3 days to different concentrations of S-DFO iron chelator. After 24 hours samples were challenged with iron. Optical density was taken at 0, 2, 20, 24, 48 and 72 hours.

**Results:** The results of the experiment demonstrated that gram-positive bacteria are more susceptible to the bacterial growth inhibitor S-DFO iron chelator. Furthermore, the investigation showed that the new iron chelator Starch linked DFO had a lower inhibitory effect on bacterial growth than DFO.

**Conclusions:** The results of this investigation demonstrated the effectiveness of DFO in stopping the growth of bacteria and the significance of iron for bacteria. Because new iron chelators have potent bacteriostatic qualities and shield against clinically significant bacterial contamination, they will enhance the safety of platelet transfusions.

### Emergency Nursing Didactics – Opinions, Resource, and Skill Exchange (E.N.D.O.R.S.E) – A collaborative academic initiative in the Emergency Department: Presenting Author: Dr. Mohammad Anzal Rehman

Specialist Emergency Physician Mediclinic City Hospital, Dubai

Elrasheed Salih

*Specialist Emergency Physician, Accident & Emergency Department, Mediclinic City Hospital, Dubai*

*Elrasheed.salih@mediclinic.ae*

**Abstract:** Emergency Physicians and Nurses are not often afforded opportunities to debrief and share educational knowledge surrounding decisions, implementation, and protocols.

A novel academic initiative sought to bridge identified knowledge gaps through debriefing and knowledge exchange between ED Physicians and nurses.

**Objective:** We aimed to provide a platform within the ED to promote interpersonal communication and feedback between healthcare providers, with opportunities to improve skills, confidence and standardize workflows around essential procedures/protocols.

**Methods:** Structured 30-minute sessions, supplemented by customized information handouts, were conducted monthly, with in-person and virtual teaching set up within the ED, recorded for later review. Topics included waveform capnography, Inotrope infusion settings, SVT management, Right/posterior ECG placement, high-risk pattern recognition, and Intubation workflow.

Questionnaires were utilized to determine knowledge reception, ideas on improvement, and future direction of E.N.D.O.R.S.E. sessions.

**Results:** Post-session questionnaires revealed that 92.3% of participants found the sessions provided them with new information, with approximately 88% of them ‘Very confident’ (5/5) in using the skills taught thereafter.

77% of participants rated the quality of the sessions as ‘Excellent’ (5/5), and 88% indicated they would like for the topics to be repeated in future sessions. Participants also reported that sessions offered them a safe, structured environment within which to ask questions.

**Conclusion:** Creation of a safe, structured learning environment allows ED healthcare providers to identify knowledge and skill gaps that may otherwise not be unmasked. Collaborative learning allows for ‘practiced proficiency’ of skills and offers an opportunity to enhance ED workflow and patient care.

### In-Patient Diabetes Care Link Nurse Team: The Effect of Health Education on Patient Glycaemic Control during Hospitalization and Post-Discharge: Presenting Author: Ms. Wafa Harbaji

Diabetes Educator, Endocrinology Mediclinic Airport Road Hospital, Abu Dhabi

***Thabet Rawy*** ^***2***^

*Academic associate, The British University in Dubai, UAE, rawy.sabet@buid.ac.ae*

**ABSTRACT**

Acute chronic DM complications are recurrent among hospitalized patients, which prolongs hospitalization and hinders treatment. Objectives: to measure the impact of the in-patient care link nurse team on improving diabetes care inside the hospital and post-discharge. Methods: A team of 12 staff nurses from each hospital unit at MAIR Hospital, UAE, was formed. A workshop for different in-patient diabetes care topics was presented to expand their understanding of concepts and skills. Then, they implemented the new knowledge and skills in a hospital setting. The study used quantitative measures to evaluate the improvement in diabetes care: (n = 70) patients from November to March 2023 before establishing the care link team and the next five months (n = 70) from April to August 2023 were included in the study. The first group received structured diabetes education by a certified diabetes educator and conventional in-hospital nursing care. Whereas the second group received structured diabetes education and continuous follow-up during admission by the care link team. After discharge, both groups HbA1c were monitored regularly. Results for both groups showed significant improvement in HbA1c after receiving the structural diabetes education (*P* < 0.001). The rate of hypoglycemia, hyperglycemia episodes, and hospitalizations was significantly reduced. The use of insulin fine needles and blood ketone strips started regularly. The infection rate showed no significant changes. The referral rate to multidisciplinary teams has increased. Conclusions: Continuous diabetes care during hospitalization by a skilled team is crucial to achieving optimal glycemic control during hospitalization and after discharge.

### Factors Influencing the Activation of Code Blue in Nurses and Ways to Improve Nurses Satisfaction: Presenting Author: Ms. Shazia Naz

Unit Manager Mediclinic Al Jowhara Hospital, Al Ain

**Background:** Code blue is a term used by the whole world hospitals to tell cardiac or respiratory arrest emergency state conditions. This code was first introduced around the 1990s in Bethany Medical Center, Kansas. The purpose of a code blue is to intervene in cardiac-arrested or critically ill patients administered by the code blue team at hospitals. It’s no secret that resuscitation is one of the most stressful, high-stakes events nurses experience in the hospital setting. When a patient goes into cardiac or respiratory arrest, time is critical, delays to intervention are costly, and the margin for error is minimal. It’s easy to see how Code Blues can become a source of frustration and dissatisfaction for staff.

**Aim:** This research aims to analyze the nurses’ correlating factors in making the blue code activation at hospitals and find the way to empower the nurses.

**Method:** The review of the literature was conducted using the American Journal of Nursing, Allied Health Literature and PubMed databases with the search terms: nursing knowledge, nursing confidence, code blue activation, code blue, ACLS, mock codes, simulation, in-hospital cardiac arrests, and patient outcomes. The search terms generated over 100 articles. Articles that were not related to in-hospital cardiac arrest, organizational culture, code blue training, and nursing perspectives about cardiac arrest resuscitation were eliminated with 15 articles ultimately included.

**Result:** The nurses in activating the code blue team are depended on their skills in making a decision based on their cognition and experience to prevent further complications and mortality. Delayed recognition of cardiac arrest, or failure to recognize and intervene early in the management of a patient who is deteriorating, is a leading cause of morbidity and mortality because each minute of delay in receiving treatment reduces patient survival by 10%.Other concerning factors are organizational culture, unsupported organizational culture for nurses to activate the code blue team influences decision-making confidence. Organizational culture also functions as a guideline. When a decision-making process does not align with the organizational spirit, then the decision cannot run maximally. This researches showed the educational factor influenced the nurses’ decision making in activating the code-blue team. Cognitive management has an essential implication for decision-making in various situations. The obtained knowledge from various sources, either from experience or other sources, could be based on decision-making.

**Conclusion:** Regular participation in mock codes has resulted in increased nurses confidence level as well as patient structured debriefings post event have been shown to drastically improvement in team performance and early activation of the code.These debriefings often include audiovisual tools to demonstrate chest compression fraction, time to defibrillation, and other areas for improvement. Ultimately Nurse Knowledge needs to be improved to optimize the decision of code blue activation.

### Professionalism-training in undergraduate medical education in a multi-cultural, multi-ethnic setting in the Gulf Region: an exploration of reflective essays: Presenting Author: Dr. Rasha Buhumaid

Vice Dean of Graduate Medical Education, Mohammed Bin Rashid University of Medicine and Health Sciences (MBRU), Dubai

**Co-Authors: Farah Otaki**

*Strategy and Institutional Excellence, Mohammed Bin Rashid University of Medicine and Health Sciences, Dubai, United Arab Emirates. farah.otaki@mbru.ac.ae*

*Care and Public Health Research Institute (CAPHRI), Faculty of Health, Medicine, and Life Sciences, Maastricht University, Maastricht, The Netherlands*

**Katarzyna Czabanowska**

*Care and Public Health Research Institute (CAPHRI), Faculty of Health, Medicine, and Life Sciences, Maastricht University, Maastricht, The Netherland. kasia.czabanowska@maastrichtuniversity.nl*

*Department of Health Policy Management, Faculty of Health Care, Institute of Public Health, Jagiellonian University, Kraków, Poland.*

**Adrian Stanley**

*College of Medicine, Mohammed Bin Rashid University of Medicine and Health Sciences, Dubai, United Arab Emirates. adrian.stanley@mbru.ac.ae*

**Mutairu Ezimokhai**

*College of Medicine, Mohammed Bin Rashid University of Medicine and Health Sciences, Dubai, United Arab Emirates. mutairu.ezimokhai@mbru.ac.ae*

**Lisa Jackson**

*College of Medicine, Mohammed Bin Rashid University of Medicine and Health Sciences, Dubai, United Arab Emirates. lisa.jackson@mbru.ac.ae*

**Samuel B. Ho**

*College of Medicine, Mohammed Bin Rashid University of Medicine and Health Sciences, Dubai, United Arab Emirates. samuel.ho@mbru.ac.ae*

**Abstract**

**Objective**: Despite the established need to prioritize professionalism-training in developing future physicians, very few medical programs in the Gulf Region embed in their curricula discrete contextualized courses aimed at developing the corresponding competencies, while fostering self-directed learning. This study aims at exploring the perception of undergraduate medical students in a multi-cultural and multi-ethnic setting regarding their understanding of, and personal experience with professionalism through their engagement with the content of an innovative curriculum-based professionalism course, offered at a Medical School in Dubai, UAE.

**Methods**: The study used a qualitative phenomenological research design. Out of 33 students, 29 students had submitted reflective essays. The content of these essays was inductively analyzed following a six-step framework for conducting thematic analysis. The framework’s steps include familiarizing oneself with the data, generating initial codes, searching for themes, reviewing themes, defining and naming themes, and producing the report.

**Results**: The inductive qualitative analysis generated the *Professionalism Learning Journey* model. This conceptual model includes four interconnected themes: *Awareness, Acknowledgement, Realization, and Application.* The generated model depicts the trajectory that the learners appear to experience while they are engaging with the content of the course.

**Conclusion**: Integrating a professionalism-training course into undergraduate medical curriculum is likely to be positively appraised by the learners. It raises their awareness, enables them to value the subject matter and the sophistication of its application, and empowers them to put into practice the taught principles, on an individual basis and collectively. This is especially true when the course is entrenched in constructivism experiential learning theory and designed to foster self-directed learning. The introduced conceptual model, in conjunction with the innovative professionalism-training course curriculum, can serve as a template for other competencies and other schools.

### Retrospective Analysis of Management and Outcome of Preterm Premature Rupture of Membranes (PPROM) in Mediclinic Al Noor Hospital.: Presenting Author: Dr. Hala Abdeldafie

Medical Intern, Mediclinic Al Noor Hospital, Abu Dhabi

**Co –Authors: Rimpi Datta**

*Specialist Obstetrician & Gynaecologist, Mediclinic Al Noor, Rimpi.Datta@mediclinic.ae*

**Saumitra Sengupta**

*Consultant Obstetrician & Gynaecologist, Mediclinic Al Noor, Saumitra.Sengupta@mediclinic.ae*

**Objective:** A pregnancy complicated by Preterm Premature Rupture of Membranes (PPROM) is a significant challenge for healthcare professionals. However, good antenatal managements, adherence to guidelines and multidisciplinary team approach improve the neonatal and maternal outcomes.

**Methods:** We did a retrospective analysis of data from electronic medical records of all women with PPROM of membranes who delivered between December ‘21 and November ’23. Diagnosis of PPROM was suspected when women attended hospital with history of vaginal leaking before 37 weeks. The data was analysed according to period of gestation, mode of diagnosis, antibiotics prophylaxis, steroids, and admission to Neonatal Intensive Care Unit (NICU), signs of sepsis of baby and mother.

**Results**: There were 1437 deliveries, of which 39 cases had Preterm Premature Rupture of Membranes. The mean gestational age was 34.49 ± 2.2 weeks. Diagnosis was made on all patients through history and examination. Almost all patients were examined vaginally (95%), received a course of antibiotics (88%), corticosteroid was given in 76%, induced or augmented in 42% cases. Vaginal delivery was achieved in 64%. All the babies were admitted to Neonatal Intensive Care Unit due to prematurity with 100% survival. One baby (2%) showed sign of sepsis from blood culture. There was no maternal morbidity.

**Conclusion:** In our cohort of this high-risk pregnancies, the outcomes of babies and mothers were very satisfactory. Our recommendations from this retrospective small observational study would be to improve documentation, adherence to antibiotics guidelines, and more expectant management to minimise NICU admission where appropriate.

### Characterization of the expression of pain receptors in a preclinical model of statin induced myalgia: Presenting Author: Dr. Asha Cyril

Post-Doctoral Research Fellow Mohammed Bin Rashid University of Medicine and Health Sciences (MBRU), Dubai

**Co-Author: Rajan Radhakrishnan**

*Professor of Pharmacology, College of Medicine, Mohammed Bin Rashid University of Medicine and Health Sciences*

*rajan.radhakrishnan@mbru.ac.ae*

**Abstract**

**Objectives:** Statins are commonly used to lower cholesterol but often lead to side effects like myalgia, which can result in patient non-compliance. This study aimed to investigate the occurrence of muscle pain (myalgia) and fatigue induced by chronic statin treatment in rats, comparing lipophilic (simvastatin) and hydrophilic (rosuvastatin) statins. We also explored the neurobiological mechanisms by analyzing the expression of pain-transducing ion channels (ASIC3, TRPV1, and P2X3).

**Methods:** Three groups of 30 male Wistar rats each were administered simvastatin (50 mg/kg), rosuvastatin (50 mg/kg), or vehicle control for 30 days. Mechanical threshold and endurance were assessed using modified Randall-Sellito method and treadmill tests. Pain channel expression in L4 and L5 dorsal root ganglia (DRGs) and gastrocnemius muscles was studied through immunohistochemistry (IHC) and RT-PCR.

**Results:** Rats receiving high-dose statin treatment exhibited significantly reduced mechanical thresholds, notably with rosuvastatin (p=0.0014). Simvastatin-treated rats displayed greater fatigue compared to both control and rosuvastatin-treated rats (p=0.042). RT-PCR results indicated higher pain receptor expression in rosuvastatin-treated rats than controls in L4 and L5 DRGs (TRPV1 p=0.036; P2X3 p=0.048; ASIC3 p=0.02). Fluorescent IHC also demonstrated elevated pain channel expression in simvastatin-treated rats compared to controls.

**Conclusions:** This study implies that chronic statin administration leads to fatigue and lowered mechanical thresholds in rats. The increased expression of pain transducers in DRGs suggests their substantial involvement in statin-induced myalgia. This preclinical rodent model can be used to discover newer drugs that can relieve statin-induced myalgia thus reducing the non-compliance to statins in patients due to muscle pain.

### Semaglutide Treatment in Type 2 Diabetes: Evaluating Effects on Weight, Glycemic Control, and Liver Enzymes: Presenting Author: Dr. Nisrine Alghazal,

Specialist Endocrinologist, Mediclinic Dubai Mall, Dubai

**Co-Authors: Sara El Ghandour**

*Department of Endocrinology, Mediclinic Parkview Hospital*

*Adjunct Associate professor MBRU*

*Sara.ghandour@mediclinic.ae*

**Shreya Pilai**

*Quality Data Analyst, Mediclinic Middle east*

**Abstract:** This study assesses semaglutide’s impact on HbA1c, weight, and liver enzymes (AST, ALT) in 50 type 2 diabetes patients over 12 months. Semaglutide yielded significant 5% weight reduction at 12 months and reduced HbA1c (-0.15% at 6 months, p<0.001; -0.14% at 12 months, p<0.001). However, no significant change in AST and ALT levels was observed, possibly due to the absence of baseline fatty liver disease.

**Introduction:** Type 2 diabetes, obesity, and fatty liver disease collectively contribute to the metabolic syndrome, prompting exploration of novel treatments. Semaglutide, a weekly GLP-1 agonist, has shown promise in improving various metabolic parameters. This study aims to comprehensively evaluate its effects on weight, glycemic control, and liver enzymes.

**Methods:** Fifty type 2 diabetes patients initiated on semaglutide were followed for 12 months. Measurements of HbA1c, weight, AST, and ALT were recorded at baseline, 6 months, and 12 months, providing insights into the treatment’s efficacy.

**Results:** Semaglutide demonstrated significant weight reduction (5% at 12 months) and reduced HbA1c (-0.15% at 6 months, p<0.001; -0.14% at 12 months, p<0.001). However, no significant change in AST and ALT levels was observed, possibly due to the absence of baseline fatty liver disease.

**Conclusion:** Semaglutide exhibits significant benefits in weight and glycemic control but does not significantly impact liver enzymes in this study, possibly due to the absence of baseline fatty liver conditions. Further research is needed to elucidate the full spectrum of semaglutide’s effects on liver health.

### Effect of Tirzepatide on weight and metabolic parameter in diabetic and non-diabetic adults: Presenting Author: Dr. Sara El Ghandour

Consultant Endocrinologist, Mediclinic Parkview Hospital, Dubai

*Maryam Al Saeed, Rabia Cherquaoui, Nisrine AlGhazal,Shreya Pillai,Georges Hajje,Samuel Ho,Fatemeh Akbarpoor, Khadija Aakef, Madeeha Kalsekar,Sara Vidha, Mona Jomaa, Farah AlAbed, Momina Malik, Prisha Bhatia*

**Abstract:** Tirzepatide, a novel GLP-1 receptor agonist, shows promise in treating type 2 diabetes and inducing weight loss. This study evaluates its effects on weight and metabolic parameters in patients with or without type 2 diabetes, including HbA1c reduction, waist circumference, and lipid parameters.

**Introduction**: Tirzepatide, a dual-action molecule on GIP and GLP-1 receptors, enhances insulin secretion and reduces glucose concentrations. Despite its efficacy in diabetic adults, its broader impact on metabolic parameters in both diabetic and non-diabetic adults is underexplored.

**Objective:** The primary goal is to assess tirzepatide’s effects on weight loss and metabolic parameters in patients with or without type 2 diabetes over 1, 3, and/or 6 months. Secondary objectives include evaluating HbA1c reduction, waist circumference, lipid parameters, and liver enzymes.

**Methods & Results**: The cross-sectional retrospective study included 426 patients prescribed tirzepatide for at least 3 months. Significant weight reductions occurred at 1 month (2% decrease), 3 months (4% decrease), and 6 months (5% decrease), with an overall 11% decrease in weight (10 kg average loss in 6 months). Notably, diabetic patients experienced a significant reduction in AST and ALT levels compared to non-diabetics.

**Conclusion**: This study provides comprehensive insights into tirzepatide’s effectiveness and safety in diverse populations, highlighting its potential in managing metabolic parameters and promoting weight loss in both diabetic and non-diabetic individuals.

### Renal Resistive Indices: Presenting Author: Dr. Chadia Beaini

Consultant Nephrologist, Mediclinic Welcare Hospital, Dubai

**Background:** Renal resistive indices (RRI) have been shown to predict the progression of kidney disease. This study aims to evaluate the association of RRI with mortality and dialysis initiation after adjustment to therapeutic and life style interventions.

**Methods:** This is a retrospective study that included all chronic kidney disease patients followed for at least two years in three nephrology clinics between 2006 and 2019 and who had a RRI level in their files. Kaplan Meier and log rank test compared the survival of patients with normal versus high RRI. Cox regression analysis evaluated the association between RRI and death or dialysis initiation after adjustment to treatments and life style modifications.

**Results:** A total of 192 patients were analyzed: 68 had RRI < 0.7 and 124 had RRI ≥ 0.7. Their mean age was 66.5 ± 13.1 years at first visit, 78.1% were males. There was a negative correlation between baseline eGFR and RRI (p < 0.001; Spearman correlation coefficient = -0.521). The survival was significantly better in patients with RRI < 0.7 with a Log Rank test < 0.001. The univariate cox regression analysis showed a significant association between RRI and mortality (HR = 1.08; 95%CI: 1.04-1.11; p < 0.001) that remained significant after adjustment to cardiovascular risk factors and interventions such as salt reduction, blood pressure control, statins and RAAS inhibitors (HR = 1.04; 95%CI: 1.00-1.08; p = 0.036). Cox regression analysis showed a significant association between RRI and dialysis initiation (HR = 1.06; 95%CI 1.01-1.10; p = 0.011).

**Conclusion:** Our study revealed that patients with an elevated RRI ≥ 0.7 are at a higher risk of mortality after adjustment to medications and lifestyle modifications. RRI can, according to this study, be considered as an independent prognostic factor in CKD patients.

**Keywords:** Chronic Kidney Disease; Hemodialysis; Life style modifications; Mortality; Renal resistive index.

## POSTER PRESENTATION ABSTRACTS

### Apixaban in Osteoarthritis: A Multifaceted Approach through Factor Xa Inhibition and Beyond

Shirin Jannati^1^,Rajashree Patnaik^1^, Riah Lee Varghese^1^, Yajnavalka Banerjee^1,^*

^*1*^ *Banerjee Research Group, College of Medicine and Health Sciences, Mohammed Bin Rashid University of Medicine and Health Sciences (MBRU), Dubai, United Arab Emirates.*

*Address correspondence to: Yajnavalka.Banerjee@mbru.ac.ae

**Abstract:** Recent research has established a connection between vitamin K antagonists (VKAs) and an augmented risk of osteoarthritis (OA) progression, advocating for non-vitamin K oral anticoagulants (NOACs) as a safer alternative. This study explores the anti-inflammatory potential of Apixaban, a Factor Xa (FXa) targeting NOAC, in a 3D proinflammatory chondrocyte model of OA. Our findings reveal a dose-dependent mitigation of FXa-mediated inflammation by Apixaban, evidenced by reduced TNF-alpha and IL-1Beta levels, as confirmed through ELISA and qPCR analyses. Additionally, Apixaban positively influenced chondrocyte integrity, indicated by alterations in MCP-1 and aggrecan levels. Moreover, Apixaban downregulated of ERK (extracellular signal-regulated kinases)1/2 expression post-FXa treatment, suggesting a mechanism for FXa’s impact on inflammatory pathways, a result supported by Western Blot analysis. Furthermore, our study identified Apixaban’s capacity to inhibit FXa-mediated upregulation of RANKL (Receptor Activator of Nuclear Factor κ B Ligand) and its receptor RANK, implicating its role in modulating bone remodeling processes disrupted in OA. Rhodamine staining validated Apixaban’s efficacy in rescuing chondrocytes from FXa-induced alterations, emphasizing its protective role in maintaining chondrocyte health, including the stabilization of mitochondrial function and reduction in reactive oxygen species (ROS) production.

Crucially, Apixaban downregulated Protease-Activated Receptor-2 (PAR-2) expression, highlighting its targeted therapeutic action in OA by mitigating FXa-induced inflammation. Single-cell transcriptomics is currently being employed to investigate cellular responses to Apixaban, unraveling diverse cell populations and molecular pathways affected by FXa inhibition. These advancements in understanding Apixaban’s multi-layered impact could significantly influence drug development, personalized medicine, marking a transformative stride in OA treatment and management.

### Biological Indicators in Relation to Cow’s Milk: How to Determine the Resolution of Allergy?

Dr. Carlos Sanchez Salguero

*Pediatric Allergist and Pediatric Consultant*

*Mediclinic City Hospital*

**Objective:** The treatment of patients allergic to cow’s milk has evolved in recent years with oral food immunotherapy techniques supported by the first official OIT guide published. Once tolerance is achieved, these patients should maintain a daily intake of at least 240 ml of milk. However, there are no biomarkers available to determine which patients have overcome tolerance and in whom the allergy has disappeared, allowing them to transition to a milk-free diet. The evolution of milk protein levels in these patients could serve as a reference for this determination.

**Materials and Methods:** A retrospective study included all patients undergoing OIT to milk (10 patients) between the years 2019-2023, aged 5-16 years. Values of alpha-lactalbumin (ALA), beta-lactoglobulin (BLG), and casein (CAS) were determined with pre-OIT and 6m-1-2-3-4-5 years post-OIT samples. Data from the determinations over time are available for those who started in 2019-20.

**Results:** The application of the SSPS program shows average values for each protein with comparisons at different time stages. No statistical differences are observed in pre-OIT values and 6 months post-OIT (34.2-33.6; 38.1-37; 41.9-41.3, p>0.05). Comparisons were made pre-OIT-2-4-5 years, 2-4-5 years, pre-OIT-5 years, and 2-5 years, showing a progressive decrease in the values of each protein, especially pre-OIT-5 years (34.2-38.1-41.9vs3.9-5.3-6.8, CI 95%, p<0.01) and 2-5 years (19.9-23-25.6vs3.9-5.3-6.8).

**Conclusions:** In oral food immunotherapy, once the desensitization phase is completed and after 5 years of daily milk intake, there is still no data supporting which children can transition to a free diet. This study evaluates the possibility of stopping dairy intake and inducing tolerance afterward.

### EVALUATING MAJOR CARDIOVASCULAR RISK FACTORS INVOLVED IN EARLY ONSET CORONARY ARTERY DISEASE (CAD) IN MEDICLINIC WELCARE HOSPITAL (MWEL) IN DUBAI, UNITED ARAB EMIRATES: A RETROSPECTIVE STUDY, 2018-2022

Dalal Obaid

*Mohammed Bin Rashid University of Medicine, Dalal.obaid@students.mbru.ac.ae*

Malak Dawood

*Mohammed Bin Rashid University of Medicine, Malak.dawood@students.mbru.ac.ae*

**Abstract**

**Objective:** The primary objective of this study is to assess the major cardiovascular risk factors involved in the development of remature Coronary Artery Disease (CAD) for the presence of common risk factors in modern-day society which includes: hypertension, dyslipidemia, smoking history, BMI, gender and ethnicity.

**Methods:** A five-year retrospective cohort study assessing data from the Catheterization Laboratory (CATH LAB) in Mediclinic Welcare Hospital (Dubai, UAE) from 1st January 2018 until 31st December 2022. The data was used to explore major cardiovascular risk factors found among patients with documented premature CAD in men under forty-five years and women under fifty-five years.

**Results:** Results showed that out of one hundred ten patients that fit the inclusion criteria, seventy-seven men and thirty-three women. Patients were then evaluated based on the risk factors, 83.6% of the patients had dyslipidemia, 57.3% of the patients had hypertension, 42.7% of the patients were overweight (BMI between 25-29), and 43.6% of the patients were obese (BMI >30). In addition, 55.5% of the patient population was South Asian.

**Conclusion:** The findings of this study indicate the strong association of certain risk factors in the development of premature CAD. It shows that the strongest risk factors that are associated with the diseases are: dyslipidemia, male gender, hypertension, BMI of overweight or obese and South Asian ethnicities have a strong correlation to the development of the disease.

### Investigating the Impact of Automation of Free Testosterone on the Turnaround Time, Accuracy, and Cost Compared to Manual ELISA Technique: A Retrospective Study

Dr. Aisha Meskiri

Health Sciences Division Chair, Higher Colleges of Technology, Abu Dhabi, UAE, ameskiri@hct.ac.ae

Ms. Hala Issa

Clinical instructor, Higher Colleges of Technology, Abu Dhabi, UAE, hissa@hct.ac.ae

Additional authors: A. Alwahedi^1^, A. Alharthi^1^, D. Almehairbi^1^, M. Alhosani^1^, N. Ghanem^1^, E. Darwish^2^, A. Bayoumi^2^,

^1^ Abu Dhabi Colleges, Higher Colleges of Technology, Abu Dhabi, United Arab Emirates

^2^Unilabs Middle east, Dubai, United Arab Emirates

**Abstract**

**Background:** Manual Enzyme-linked immunosorbent assay (ELISA) is the most common serological assay used to measure free testosterone levels within laboratories across the world including the United Arab Emirates (UAE). Automated chemiluminescence immunoassay (CLIA) is an emerging methodology within the clinical laboratories, based on the same technical foundation as ELISA with promising improved diagnostic and clinical outcomes. In clinical laboratories, testosterone measurements are used to diagnosis multiple conditions including hypogonadism, erectile dysfunction, prostate cancer, and polycystic ovary syndrome. Efficient testing of testosterone will be improved clinical diagnosis.

**Objectives:** To compare ELISA and CLIA techniques in quantifying free testosterone, encompassing aspects such as turnaround time (TAT), accuracy, and cost-effectiveness.

**Methods:** Retrospective records from Unilabs Laboratory Information System (Dubai, UAE) of 2413 testosterone blood samples analyzed via ELISA from January 2022 till the end of June 2022 and 3831 testosterone blood samples analyzed via CLIA from January 2023 till the end of June 2023 were reviewed. After which a comparative analysis was conducted between the two techniques.

**Results:** CLIA method exhibited a significantly shorter TAT (4 hours) compared to ELISA making it more efficient in diagnosis. Both techniques demonstrated comparable levels of accuracy within the reference range. Interestingly, CLIA also proved to be more cost-effective than ELISA, with a 28% cost savings, highlighting the unexpected economic benefits of adopting CLIA.

**Conclusions:** Although there was a good analytical agreement between ELISA and CLIA, CLIA showed advantage of being automated with fast turnaround time and long-term cost-effectiveness when compared to ELISA.

### The relationship between scapular fracture and injury severity in blunt thoracic trauma

Ashraf F. Hefny

College of Medicine and Health Sciences, UAEU, Al Ain Hospital, Al ain, United Arab Emirates ahefny@uaeu.ac.ae

Sherif A. Fathi

Department of Surgery, Faculty of Medicine, October 6th University, Cairo, Egypt sherif.soliman2003@gmail.com

Nirmin A. Mansour

Department of Family Medicine, Ambulatory Health Services, Abu Dhabi health Services Company, UAE

nmansour1234@gmail.com

**Abstract**

**Objective:** Scapular fracture is a rare encounter in blunt trauma patients (0.8% - 2%). The scapula is surrounded by strong groups of muscles offering good protection for the bone that needs high energy trauma to be fractured.

We aim to study the scapular fractures and their relation to injury severity and mortality in blunt chest trauma patients.

**Methods:** We retrospectively collected data of all patients who were admitted to Al Ain Hospital with blunt chest trauma from December 2014 through January 2017. The Injury details of all blunt chest trauma patients were retrieved from Al Ain hospital trauma registry.

**Results:** During the study period, there were 4779 patients included in the Trauma Registry of Al-Ain Hospital, 669 (13.9%) patients had blunt chest trauma. Scapular fracture was present in 29 (4.3%) of blunt chest trauma patients, Injury severity parameters as ISS, NIS, and ICU admission were significantly higher in patients with scapular fracture compared to other patients without fracture. Overall mortality in patients with scapular fracture was 9.6% compared with 13 (2%) patients in patients without scapular fracture which was not statistically significant (p=0.134 Fishers exact test).

**Conclusions:** Scapular fracture in blunt chest trauma patients indicates high energy type of trauma. Particular attention and meticulous evaluation should be paid to head and neck injuries to avoid missing injuries.

### Value of the clinical pharmacist interventions in the application of the American College of Cardiology (ACC/AHA) 2018 guideline for cholesterol management

Mohammad M. AlAhmad.

Assistant Professor, Al Ain Pharmacy College, mohammad.alahmad@aau.ac.ae.

Khozama AlAhmad,

Senior Clinical Pharmacist, Mediclinic AlAin Hospital, Khuzama.Ahmed@mediclinic.ae.

Published: March 27, 2023 in PLOS ONE journalhttps://doi.org/10.1371/journal.pone.0283369

**Abstract:** Historically, we saw the NCEP and their published recommendations regarding Elevated blood levels of lipoproteins (cholesterol, triglycerides, phospholipids) which basically the major cause of atherosclerosis and atherosclerosis-induced conditions, such as coronary heart disease (CHD), ischemic cerebrovascular disease, and peripheral vascular disease. Since then, we are using specific LDL-C and/or non–high-density lipoprotein cholesterol (non–HDL-C) treatment targets when we are managing our patients.

**Objective:** The study aims to examine the extent to which the updated ACC/AHA management of blood cholesterol guideline (2018) is implemented in practice and to assess the value of the clinical pharmacist interventions in improving physicians’ adherence the guidelines recommendations.

**Methods:** We utilized in this study an interventional before-after design. The study was conducted on 272 adult patients who visited the study site internal medicine clinics and were candidates for statin therapy based on the 2018 ACC/AHA guidelines for cholesterol management. Adherence to guideline recommendations was measured before and after clinical pharmacists’ interventions by calculating the percentage of patients receiving statin therapy as per guideline recommendation, the type and intensity (moderate or high intensity) of statin therapy used, and the need for additional non-statin therapy.

**Results:** Adherence with guideline recommendations was significantly improved from 60.3% to 92.6% (X2 = 79.1, p = 0.0001) after clinical pharmacist interventions. Among patients who were on statin therapy, the percentage of those who were on proper statin intensity increased significantly from 47.6% to 94.4% (X2 = 72.5, p = 0.0001). The combination of statins with non-statin therapies such as ezetimibe and PCSK9 inhibitors increased from 8.5% to 30.6% (X2 = 95, p<0.0001) and from 0.0% to 1.6% (X2 = 6, p = 0.014), respectively. The use of other lipid-lowering agents was diminished from 14.6% to 3.2% (X2 = 19.2, p<0.0001).

**Conclusions:** Collaboration between physicians and clinical pharmacists is a crucial strategy to improve patients’ treatment and hence, achieve better health outcomes among patients suffering from dyslipidemia.

### Dysregulation of iron homeostasis in mice with experimental autoimmune encephalitis

Mohammad G. Mohammad*, Ahmad M. Al-Rawi, Dana Salahat, Salam Dakalbab, Ameera Abu-Qiyas, Shahad Arikat, Abdul Soofi, Jibran S Muhammad, Mawieh Hamad

**Abstract**

**Background** Multiple sclerosis (MS) is a chronic neuroinflammatory and degenerative disease of the central nervous system (CNS) caused by cell-mediated immune response that is associated with demyelination and axonal degeneration, as well as damage to myelin-producing oligodendrocytes. MS is increasingly linked to dysregulation of iron metabolism and its overload cytotoxicity. Disrupted iron metabolism may lead to toxic accumulation and consequential damage to neuronal cells.

**Objectives** Pathological evidence suggests iron regulatory proteins are altered in different tissues during the course of MS, as well as in specific anatomical areas of the brain.

**Methods:** Mice with Experimental Autoimmune Encephalitis (EAE) at peak disease were used to investigate the distribution of iron deposits and the related proteins in CNS and systemic compartments during neuroinflammation. Iron deposits were characterized using Prussian blue iron stain. Proteins regulating iron metabolism including Hepcidin, HIF-1, IRP-1, ferroportin, ferritin, transferrin and transferrin receptor were characterized using western blotting.

**Results:** Iron deposits characterization using Prussian blue stain revealed accumulation of iron in spinal cord, meninges, olfactory bulb, cerebellum but not corpus callosum and choroid plexus. Iron accumulation also observed in livers and spleens from EAE mice. Altered levels of iron-regulating proteins were also observed. Iron homeostasis regulatory proteins, Hepcidin, IRP-1 and HIF-1 were upregulated in all tissues analyzed in EAE mice. Similarly, increased levels of ferritin were detected in all tested organs with the exception of brain parenchyma. Consistently, increased levels of transferrin receptor and reduced ferroportin levels were observed in olfactory bulb, spinal cord and spleen.

**Conclusions:** Taken together, these findings clearly demonstrate that EAE associates with disrupted iron homeostasis. Further studies are needed to ascertain the molecular regulatory mechanisms underlying iron metabolism dysregulation and its bearing on the pathogenesis of neuroinflammation.

### Role of maternal gut microbiota and obstetric complications: A literature review

Dr. Fatema Albafta

Dubai health, General Surgery Resident, fatema.albafta@residents.mbru.ac.ae

Rida Maryum Mohammed Bin Rashid

University of Medicine and Health Sciences, College of Medicine, Medical student, rida.maryum@students.mbru.ac.ae

Maryam Hashi Mohammed Bin Rashid

University of Medicine and Health Sciences, College of Medicine, Medical student, maryam.hashi@students.mbru.ac.ae

Sharon Nandkishore Mohammed Bin Rashid

University of Medicine and Health Sciences, College of Medicine, Medical student, sharon.nandkishore@students.mbru.ac.ae

Dr. Alessandra Pipan

Mediclinic City Hospital, MD, Master in Medical Anthropology, Italian Board Certified (OB/GYN, Management of Health Services), RCOG, alessandra.pipan@mediclinic.ae

**Abstract:** The composite and elusive role played by the microbiome in orchestrating complex molecular network in the human body has long been investigated. Mounting evidence suggests that alteration of this microbiota plays a role in major obstetric complications.

**Objective:** This state-of-the-art literature review focuses on investigating the association between human gut microbiome with four obstetric complications, namely, preterm labor (PL), preeclampsia (PE), gestational diabetes (GDM), and recurrent pregnancy losses (RPL). This research addresses the limitations in existing knowledge, testing methodologies, molecular pathways and confounding variables like environmental factors, while also providing suggestions for future advancements.

**Methods:** Four independent reviewers conducted a literature search on PubMed, MEDLINE, and Google Scholar databases, for papers published in English from 2013 to 2023. 34 articles (4 PL, 11 PE, 10 GDM, 9 RPL) fit the inclusion criteria.

**Results:** Aberrant gut microbiome composition and function is associated with RPL as it induces a proinflammatory state thereby directly affecting the immune system. Similarly, studies show that in GDM, there is presence of chronic inflammation due to disruption of intestinal mucosal barrier by LPS producing bacteria. Subsequently, reduced levels of clostridium bacteria and butyrate producing bacteria are seen in PL and PE respectively. Studies show that alterations in the gut microbiota during pregnancy contribute to changes in maternal immune system and metabolism leading to various complications.

**Conclusions:** The application of artificial intelligence in assembling and cross matching metagenomic, transcriptomic and metabolomic data, while simultaneous correlating the structural and functional aspects holds promising prospects.

### Qualitative study undertaken in MNOO evaluating the awareness of fetal movement information provided to pregnant women.

Dr Rimpi Datta Specialist Ob-Gyn,

Mediclinic Al Noor Hospital, Abu Dhabi, email: rimpi.datta@mediclinic.ae

Dr. Rawan Al Shurafaa Medical Intern,

Mediclinic Al Noor Hospital, Abu Dhabi., email: rawan.omar321@hotmail.com

Dr. Raoya Farah Consultant and HOD Ob Gyn,

Mediclinic Al Noor Hospital, Abu Dhabi, email: raoya.farah@mediclinic.ae

Dr Iram Siddiqui

Consultant Ob Gyn, fetal medicine specialist, Mediclinic Al Noor Hospital, Abu Dhabi, email: iram.siddiqui@mediclinic.ae

**Background:** Reduced fetal movement can be an important symptom of fetal compromise. Maternal perception of fetal movement is one of the first signs of fetal life and is regarded as a manifestation of fetal well-being. A significant reduction or sudden alteration in fetal movements is a potentially important clinical sign and can be a concern for both the mother and obstetrician. Studies have shown a 2 fold increase in maternal perception of reduced fetal movements 2 weeks prior to still birth.

**Aim:** We undertake a qualitative study to review the information given to pregnant women on fetal movements during the pregnancy. We assessed the type of information given- verbal or written, their understanding of the information, when and how to access the health services when faced with altered fetal movements and the risk factors that are linked with higher perinatal morbidity and mortality.

**Method:** Questionnaire were sent out to a group of randomly selected 100 pregnant women of 13 nationalities who were more than 26 weeks of gestation, attending Mediclinic Al Noor Hospital outpatient obstetric clinic. The Data was analyzed to review the quality of information given, pregnancy risk factors and factors contributing to the non-reporting of the reduced fetal movements.

**Discussion and results:** After analyzing the data it was noted that 26% of women noted reduced fetal movements, out of which 17% chose to wait at home and the fetal movements became normal, 3% chose to reach their physicians through email or phone call, 4% reported to the hospital and adequate management according to the RCOG guidelines were followed by the medical staff and 2% did nothing about it, the reason being busy with household chores(1%) and ignoring and assuming it is normal (1%). Also a gap was noted in the information provided to patient with no verbal information in 10% of cases and no written information in 37% of cases. It was found during the analysis that we have a large number of high risk pregnancy in our population (52%) with diabetes being the most prevalent (21%) who need more attention and awareness. All the women in the study group had regular antenatal check-up.

**Conclusion:** We recommend regular audit to ensure guidelines are met on patient’s awareness and education on fetal movements so as to reduce the barriers for underreporting of the condition. We need to bridge the gap in the verbal and written information provided to pregnant women by encouraging nurses and physicians working in obstetric outpatient areas to discuss about fetal movements at every point of contact with them. Special attention to be given to the high risk obstetric category to reduce fetal morbidity and mortality.

### Antibiotic stewardship program: Barriers and enablers of implementation in primary health care in Emirates Health Services, United Arab Emirates.

Dr. Shaza Shaaban Zackaria

Family medicine specialist, PRIVATE SECTOR, DHA

Dr. Hiba Rabie Mohammed

Family medicine specialist, PHCC, UAQ, EHS

**Abstract:** Antimicrobial stewardship programs aim at promoting the appropriate use of antibiotics. It is applied in UAE hospitals and primary healthcare settings to overcome the increasing antibiotic resistance. We have not come across any study regarding the barriers to antibiotic stewardship implementation in PHCC.

**Objective:** To explore these challenges and facilitators from the perspective of healthcare workers that might help build a strategic plan to enable healthcare workers to implement an antibiotic stewardship program.

**Methods:** A cross-sectional study on the ASPS program in the UAE, the study was based on an electronic online questionnaire survey disturbed to all physicians and pharmacists in emirates health services PHCC, in the UAE.

**Results:** 192 responses were received from 7 primary health care centers. 85% were familiar with the concept of antimicrobial stewardship. Most participants thought that antibiotic resistance is a problem in our community and agree that the antibiotic stewardship program effectively reduces the antibiotic (89% and 86% respectively). Around 97% of the respondents from the EHS PHCCs reported that Physician education and training about antibiotic stewardship is important for the implementation of the ASPs.87% agree that non-clarity of the guidelines could be a barrier to implementation of antibiotic stewardship.

**Conclusions:** Healthcare workers highly perceived the antibiotic resistance problem, antimicrobial stewardship program, and its effectiveness. This study concluded that organizational support was the most perceived enabler of the program while the guideline-related barrier was the most perceived barrier by healthcare workers.

### Double Trouble: lessons learnt from synchronous renal tumors within a single kidney- A case report

Tariq Abdul Hamid^1^, Amr Elmekresh^1^, ^1^Urology Residents- Dubai Health, drtariq_hamid@yahoo.com, a.mekresh@yahoo.com

Ali Thwaini^2^, Amrith Rao^2^ Consultant Urologist-Mediclinic Hospital, amrith.rao@mediclinic.ae

**Abstract**

- Most prevalent solid kidney tumor (80% of cases).
- Represents about 2-3% of all adult malignancies; Clear cell (80%), Papillary (10%), Chromophobe (5%).
- Clear cell papillary Renal Cell Carcinoma (RCC) Low-grade renal tumor type, and is newly classified in the 2016 WHO renal tumor classification.
- Reports of benign and malignant renal tumors occurring simultaneously in both kidneys, with very few instances of distinct subtypes of malignant renal tumors developing within the same kidney.
- Case of a 47-year-old male, who Underwent successful robotic-assisted left adrenal-sparing radical nephrectomy and diagnosed with clear cell papillary RCC and papillary RCC in the left kidney

**Introduction**

- Accounts for 80% of all primary renal tumors.
- Limited exploration of coexistence of benign and malignant tumors in the same kidney.
- 10 to 32 percent of patients with oncocytoma may have coexisting RCC, Suggesting the possibility of two distinct RCC tumors in a single kidney.
- Index case is a 47 year old healthy male with incidental discovery of two separate lesions identified as clear cell papillary RCC and type 2 papillary RCC, respectively post Robot assisted radical left nephrectomy.

**Case Discussion**

- 47-year-old male,was Referred to Urology clinic with ultrasound findings of a complex cyst with calcification ***(Figure 1***).
- Medical history significant for epilepsy on levetiracetam 1000 mg twice daily and was seizure free, with no history of hypertension, diabetes, or ischemic heart disease.
- Unremarkable physical examination and Normal lab parameters and renal function (Hemoglobin 15,WBC 7, platelets 250, creatinine 0.9 mg/dl).
- Contrast-enhanced CT scan (CT) revealed a 4.2cm thin-walled partially exophytic lesion with calcification, with another 2.4cm left lower pole lesion with heterogeneous contrast enhancement ***(Figure 2***).
- Case was discussed in Multidisciplinary Team meeting, and Advised to undergo robot-assisted left partial nephrectomy.
- Intraoperatively, lower pole tumor identified as completely endophytic, thus Left Radical Nephrectomy with adrenal gland sparing procedure performed ***(Figure 3***).
- Histopathology revealed Cortical tumor measuring 45x40 mm identified as Type 2 papillary RCC, and Second lesion measuring 25x20 mm diagnosed as clear cell papillary RCC with negative margins and no microvascular invasion (Pathological staging: pT1b pNx pMx).
- Surveillance CT scan performed 12 weeks later showed no evidence of local or regional recurrence.

**Discussion**

- RCC constitutes a small percentage, about 2-3%, of all cancer cases. The incidence of RCC has been on the rise in recent decades, primarily due to its incidental detection.
- Risk factors for RCC include smoking, hypertension, exposure to toxic substances at work, obesity, acquired cystic kidney disease, and genetic predisposition
- Richstone et al. found that among 57 patients, 9 showed disparities in pathology between their primary and satellite tumors, with a mix of clear cell RCC and papillary RCC.
- Patel et al. discovered that there was an 89% agreement in malignancy among patients with bilateral synchronous renal tumors. However, there is no available data regarding unilateral synchronous occurrence of different types of RCC.
- The current World Health Organization (WHO) classification of renal epithelial tumors is grounded in their histopathological, immunohistochemical, and genetic characteristics ^(4)^. One such entity observed in the case under consideration is known as clear cell papillary RCC (CCPRCC).
- clear cell papillary RCC differs from both clear cell RCC and papillary RCC in histological appearance. immunohistochemical staining demonstrating strong expression of cytokerain 7 (CK7), no or low expression of CD10 and racemase and negative expression of transcription factor E3 (TFE3)
- Surgical removal (either partial nephrectomy or radical nephrectomy) is the recommended approach for all localized RCCs

**Conclusion**

- Clear cell papillary RCC and papillary RCC can coexist in a single kidney, but is rare, Genomic alterations may contribute to the simultaneous occurrence of synchronous kidney tumors in the same kidney
- CT Scan with contrast can help in proper evaluation of complex lesions in the kdineys.
- Needs extensive studies for meaningful comparisons and insights into disease progression.
- As Each RCC subtype exhibits unique characteristics, Histological subtype recognition is crucial for Prognosis, Tailoring follow-up strategies, and Designing relevant clinical trials

### Feprazone in Osteoarthritis: Enhanced Anti-Inflammatory Action, Matrix Remodeling, and PAR-2/TNF-α Pathway Modulation

Rajashree Patnaik^1^, Shirin Jannati^1^, Alaa Al-Refaie, Dana Nasrallah^2^, Nerissa Naidoo^3^, Yajnavalka Banerjee^1^*

Banerjee Research Group, College of Medicine and Health Sciences, Mohammed Bin Rashid University of Medicine and Health Sciences (MBRU), Dubai, United Arab Emirates.

Summer Internship Program, Banerjee Research Group, College of Medicine and Health Sciences, MBRU, Dubai, United Arab Emirates.

College of Medicine and Health Sciences, MBRU, Dubai, United Arab Emirates

Current address: Department of Biotechnology, University of Sharjah, Sharjah, United Arab Emirates *Address correspondence to: Yajnavalka.Banerjee@mbru.ac.ae

**Abstract:** This study addresses the need for innovative therapeutic interventions in osteoarthritis, a prevalent degenerative joint disorder. Recognizing feprazone, a nonsteroidal anti-inflammatory drug, for its efficacy in treating inflammatory conditions, including osteoarthritis, we aimed to enhance our understanding of its antiinflammatory mechanisms and explore potential synergistic interactions with Vitamin D and Curcumin. Utilizing a state-of-the-art inflammation model, we employed chondrocytes differentiated from bone marrow– derived mesenchymal stem cells in a 3D culture. Our investigation focused on feprazone’s dose-dependent suppression of PAR-2, a crucial G-protein coupled receptor in inflammation. Simultaneously, we explored the anti-inflammatory mechanisms of Vitamin D and Curcumin, particularly their regulatory effects on SOX4 and ADAMTS5 expression, along with their established roles in modulating the PAR-2 → TNF-α-induced MCP-1 and aggrecan loss pathways. Feprazone exhibited a dose-dependent attenuation of PAR-2 expression, suggesting an innovative mechanism for mitigating inflammation. Additionally, Vitamin D and Curcumin showcased potent regulatory effects on SOX4 and ADAMTS5 expression, unveiling a novel aspect of their anti-inflammatory mechanisms. Coadministration of feprazone with either Vitamin D or Curcumin demonstrated a synergistic effect, amplifying PAR-2 downregulation across various inflammatory parameters, including TNF-α-induced loss of aggrecan, proinflammatory cytokine expression, and the SOX-4/ADAMTS-5 signaling pathway. Our findings underscore feprazone’s robust anti-inflammatory mechanisms, potentially mediated through PAR-2 signaling, and highlight their potentiation in the presence of Vitamin D or Curcumin. This multi-pronged discovery presents a transformative approach in osteoarthritis management, offering the potential for optimized medication doses, minimized adverse effects, and significantly improved patient outcomes. The study’s implications contribute to advancing the understanding of innovative therapeutic strategies in osteoarthritis.

### Different Strategies for Managing Dupuytren’s Contracture: A Literature Review

Fatema AlBafta1, Ghazal Saeed1, Hind AlAwadi1

College of Medicine, Mohammed Bin Rashid University of Medicine and Health Sciences

**Introduction:**

- Dupuytren’s contracture is an abnormal thickening of tissues in the palm of the hand
- Dupuytren’s contracture is believed to be hereditary, the exact cause is not known. It may be linked to cigarette smoking, alcoholism, diabetes, nutritional deficiencies, or medicines used to treat seizures.
- According to the present study, the prevalence of Dupuytrendiseasein the world is 8.2%(95% CI 5.7–11.7%).
- There are various management approaches for this disease

**Aim:**

- This literature review aims to explore the conventional treatments for Dupuytren’s Disease (DD), including surgical and non-surgical methods, and compare patient-reported outcomes for each approach.
- The review provides insights into the current landscape of DD management, highlighting the strengths and limitations of each treatment option.
- The ultimate goal is to steer Dupuytren’s contracture research towards advancing minimally invasive treatment options and developing a management algorithm for DD patients.

**Methods:**

- A comprehensive search was conducted in PubMed, MEDLINE, and Google Scholar.
- Inclusion criteria comprised studies including both genders aged above 18 years and published within the last 5 years.
- The review covered various non-surgical approaches, surgical approaches, and adjuvant therapies.

**Results:**

32 papers were reviewed after satisfying the inclusion criteria. With that in mind, we assessed the efficacy and limitations of nonsurgical approaches, namely steroid injections, CCH injections, and radiotherapy in treating Dupuytren’s contracture and comparing them with surgical approaches like fasciectomy and needle aponeurotomy.

**Conclusion:**

- The choice of treatment for Dupuytren’s contracture should be based on a personalized care approach, taking into consideration the severity of the condition and the assessment of both the patient and physician.
- Further research is needed to advance minimally invasive treatment approaches and enhance our understanding of DD management.

### Knowledge, and Attitude towards Thrombosis and Thrombophilia among the Population of the United Arab Emirates; A Cross - Sectional Study

Lama Musallam, lmusallam@hct.ac.ae Department of Medical Laboratory Science, Faculty of Health Sciences, Higher Colleges of Technology (HCT), UAE

Fatima Rwashed, Maha Alhammadi Department of Medical Laboratory Science, Faculty of Health Sciences, Higher Colleges of Technology (HCT), UAE

**Objectives:** Venous thromboembolism (VTE) is preventable condition that has largely gone unnoticed causing significant morbidity and mortality worldwide. No studies assessing thrombophilia knowledge, while inconsistent thrombosis findings were observed in the literature. This study aimed to determine knowledge, and attitude towards thrombosis/thrombophilia among UAE population.

**Methods**: A quantitative, descriptive, cross-sectional design using online-based-survey was employed. Participants of both genders, different nationalities, aged≥18 years and from all UAE cities were included. Survey content was validated by thrombosis/thrombophilia experts, translated to Arabic, and pilot-tested. Survey included close-ended questions;(16)-demographic, (13) assessing knowledge, and (22) evaluating attitude. All statistical analysis was conducted using SPSS-Version-24-2018.*P*-value of <0.05% was statistically significant. Data was collected after receiving ethical approval from HCT.

**Results**: (974) out of (1040) participants were included, age range [18-76]; and mean 27.06 years. Subjects were mainly females(82.2%;n=801),single(69%;n=681),Emirati(79.2%;n=771),Bachelor degree holders(59.8%),unemployed(68.5%;n=667),and had low monthly income(0-5000AED)(68.7%;n=669).(58.2%;n=567)of participants had medical/health education background.(95.2%),(81.3%),(93.1%) had never, no family members/relatives, or no friends been diagnosed with thrombosis, respectively. Whereas (98.3%), (95.6%), (97%) results were found for thrombophilia. Source of knowledge on Thrombosis/Thromphophila were mostly from Educational Institutes(18.5%)(16.6%), Media(17.1%)(13.3%),HealthcareProfessionals(17.1%)(14.6%),Internet(12.8%)(9.1%),and Family/relatives(12.8%)(8.5%).Furthermore,(82.1%;n=800)had negative attitude towards thrombosis/thrombophilia although(74.125%;n=593)stated that they had heard about thrombosis(*p<*.001).Whereas(17.1%;n=167) had neutral attitude and only (0.7%)had positive attitude. Majority of participants with negative attitude were females(83%;n=664),single(68.375%;n= 547)(*P<*.001),Bachelor degree holders(60.125%;n=481),and with no medical/health education background(56.75%;n=454).

**Conclusion**: There was poor knowledge on risk factors, signs/symptoms, diagnosis, treatment, and preventive measures of Thrombosis/Thrombophilia plus negative attitude was demonstrated among UAE population. It’s recommended to organize more awareness campaigns to enhance knowledge and promote public-awareness of highly-serious yet preventable thrombotic conditions. Furthermore, more educational programs must be conducted to encourage positive attitude towards thrombosis/thrombophilia by improving population’s lifestyles/habits. Finally, it’s suggested to implement public genetic testing to help healthcare providers make better medical judgments and enhance preventative medicine.

### Improving Blood Transfusion Consent Documentation Compliance in Intensive Care Unit – A Quality Improvement Project

**Maryam Jafari**

*Mohammed Bin Rashid University of Medicine and Health Sciences, Maryam.Jafari@alumni.mbru.ac.ae*

**Noora Al Qassim**

*Mohammed Bin Rashid University of Medicine and Health Sciences, Noora.alqassim@alumni.mbru.ac.ae*

**Yusuf Hallak**

*Mohammed Bin Rashid University of Medicine and Health Sciences, Yusuf.hallak@alumni.mbru.ac.ae*

**Ahmedyar Hasan**

*Mohammed Bin Rashid University of Medicine and Health Sciences, Ahmedyar.hasan@alumni.mbru.ac.ae*

**Yasser Ghaffari**

*Mohammed Bin Rashid University of Medicine and Health Sciences, Yasser.Ghaffari@alumni.mbru.ac.ae*

**Suha Eltigani**

*Mediclinic Middle East seltigani@mubadalahealth.ae*

**Waafika Seegers**

*Mediclinic Middle east waafika.seegers@mediclinic.ae*

**Abstract**

**Objective:** To improve compliance of Blood Transfusion consent forms documentation in the patient’s medical records to 80% by March 2023.

**Methods:** Data was collected based on all blood orders in the intensive care unit. Our main outcome measure was the completeness of the blood transfusion consent form on the electronic health system for all transfused blood. The PDSA cycle was utilized to carry out this audit. We evaluated the effectiveness of each cycle’s intervention by assessing the quantitative change in blood transfusion consent compliance before and after the implementation of actions in each cycle.

**PDSA 1:** Physicians were provided data-driven feedback on their performance with regards to blood transfusion consent form compliance.

**PDSA 2:** Physicians and nurses were exposed to a flow-chart illustrating the necessary steps to undertake when ordering a blood transfusion.

**Results:** Following the implementation of individualized physician feedback in our first PDSA cycle, there was an overall increase in the rate of completion of the transfusion consent forms and in December 2022 the target compliance rate of our project was reached (82%). However, in the subsequent month, the compliance rate decreased to 36%. Following implementation of the flow chart as a second PDSA cycle, the rate of compliance was 39%. From February 2023 to March 2023, compliance rates increased further from 50% to 75%.

**Conclusions:** Our project demonstrates that the use of personal feedback (PDSA 1) as well as providing a visual aid (PDSA 2) can both play an important role in increasing awareness of compliance to the blood transfusion documentation policy.

### Reporting the effectiveness of Dietary Intervention on Weight Loss Outcomes in Patients with Obesity and Overweight: A Retrospective Chart Review at a Center of Excellence in the UAE

Mona Joumaa

Mediclinic Parkview Hospital, Dietary department, Mona.joumaa@mediclinic.ae

Dr. Sara El Ghandour

Mediclinic Parkview Hospital, Endocrinology department, Sara.ghandour@mediclinic.ae

Dana Nabeel Abdel-Rahim Sharjah University, Research Institute for Medical and Health Sciences*, dabdel-rahim@sharjah.ac.ae*

**Objective:** This retrospective study aims to assess the effectiveness of dietary intervention on weight loss outcomes in patients with obesity and overweight attending a Center of Excellence for Obesity Management in the UAE.

**Method:** Modest weight loss of 5% is recognized to prevent the progression metabolic conditions. This study, comprises 269 participants. Descriptive data analysis revealed predominantly female (64.3%) and Caucasian (81.0%).

**Results:** 5.4 % significant reductions in weight. BMI dropped significantly by 1.7%. Fat percentage reduced by 5.5%.Waist circumference reduced by5.2%. Visceral fat reduced by 24%. BMI change and waist change were positively correlated with total number of sessions. Waist change was positively correlated with length of treatment.

Thus, there was negative correlation between fat mass loss and cessation of dietician visit. Unexpectedly, this finding was offset by a positive correlation between the weight change and waist change and the cessation period of more than 3 months.

That can possibly be explained that patients who had long pauses had more time to achieve more weight loss, however at the expense of muscle loss rather than fat loss. All of which highlighting the positive impact of compliance to dietician visits.

Females experienced significant changes in fat percentage and waist circumference compared to males. Males exhibited a greater reduction in visceral fat, with a significant difference in BMI.

**Conclusion:** The dietary intervention resulted in notable improvements in anthropometrics. These findings provide insights into the efficacy of the dietary treatment. The gender-specific variations underscore the need for tailored approaches in obesity management.

### Association of carotid calcifications with cardiovascular risk factors in adults; a cross-sectional nested case-control panoramic study in Dubai

Nabi S^1^, Albreiki M^1^, Khamis AH^2^, Chaudhry J^2^

1 College of Medicine, Mohammed Bin Rashid University of Medicine and Health Sciences, Dubai, United Arab Emirates.

2 Department of Oral Diagnostics and Surgical Sciences, Hamdan Bin Mohammed College of Dental Medicine, Mohammed Bin Rashid University of

Medicine and Health Sciences, Dubai, United Arab Emirates.

**Background:**

- Ischemic heart disease is the leading cause of death in UAE
- Atherosclerosis is the most common cause of CVDs
- Panoramic radiographs are a commonly used imaging modality in dentistry
- Upper neck structures including carotid arteries are within the field-of-view of panoramic radiographs
- Carotid arteries are a common site of atherosclerosis that manifest as calcifications on panoramic radiographs
- Early detection of atherosclerosis leads to reduction in CVD morbidity and mortality

**Objective:**

- Determine prevalence of carotid calcifications in adults aged 40 and above
- Explore the association of CVD risk factors in patients with and without carotid calcifications

**Methodology:**

- The cohort were screened for carotid calcifications on panoramic radiograph
- Patients with confirmed carotid calcifications were designated as cases
- Case-control ratio of 1:3 was matched according to age, gender, and nationality
- Risk factor data was collected from the health history

**Results:**

- 811 patients reviewed
- 24 cases and 787 controls
- 488 (60%) males and 323 (40%) females
- 168 (21%) locals and 643 (79%) expatriates
- Mean age: 49 years
- Prevelance = 24/811 ≈ 3%
- Most common level of calcification: C3 vertebra
- The model test for fitness was 63.324 (df = 5)

### Evaluation of the Efficacy of Ultra-Rapid Acting Insulin Lispro-AABC in Glycemic Control in Diabetic Patients

Saif Alsaadi^1^, Omar Hamadi^1^, Zain Alnukari^1^, Dr. Maryam Alsaeed^1,2^

¹College of Medicine, Mohammed Bin Rashid University of Medicine and Health Sciences, Dubai, United Arab Emirates

²Department of Endocrinology/Diabetes, Mediclinic Hospital, Dubai, United Arab Emirates

**Background:**

- Diabetes is a chronic condition with a 19.3% prevalence in the UAE.
- Lispro-aabc (Lyumjev) is a new ultra-rapid acting insulin released in 2021.
- It has been shown to be more effective than rapid-acting insulin in reducing postprandial glucose.
- This study sought to investigate its efficacy in glycemic control in a local population.

**Objectives and Aims:** Assess glycemic control’s improvement in patients who use Lyumjev.

Compare Lyumjev’s response between type 1 and type 2 diabetes.

**Methods:**

- Study Design:
  - Retrospective Cohort Study from 2021-2023 in Dubai, UAE.
- Sample Size:
  - 58 diabetic patients using Lyumjev.
- Analysis:
  - Repeated measures t-test to evaluate Lyumjev’s efficacy.

**Results:** As shown on the bar graph, Lyumjev effectively improved glycemic control, with mean HbA1C levels showing reduction after starting the new treatment. Patients experienced a mean HbA1C reduction of 0.9, indicating a positive result of Lyumjev use.

As it stands, there is only a slight and negligible difference in terms of change of HbA1C levels between type 1 and type 2 diabetes (p value = 0.482, non-significant).

**Conclusion:** Our study shows that Lyumjev has potential for enhancing glycemic control in a diverse patient population. Its effectiveness is influenced by different factors, emphasizing the need for personalized treatment plans.

These findings offer valuable insights for healthcare providers to improve diabetes management and enhance patients’ quality of life. Further research is needed to explore Lyumjev’s efficacy and its broader application in diabetes care.

